# Recent Advances in the Development of Liquid Crystalline Nanoparticles as Drug Delivery Systems

**DOI:** 10.3390/pharmaceutics15051421

**Published:** 2023-05-06

**Authors:** Jassica S. L. Leu, Jasy J. X. Teoh, Angel L. Q. Ling, Joey Chong, Yan Shan Loo, Intan Diana Mat Azmi, Noor Idayu Zahid, Rajendran J. C. Bose, Thiagarajan Madheswaran

**Affiliations:** 1 School of Pharmacy, International Medical University, Jalan Jalil Perkasa 19, Bukit Jalil, Kuala Lumpur 57000, Selangor, Malaysia; jassica.leu@student.imu.edu.my (J.S.L.L.); angel.ling@student.imu.edu.my (A.L.Q.L.); joeychong@student.imu.edu.my (J.C.); 2 Department of Chemistry, Faculty of Science, Universiti Putra Malaysia, Serdang 43400, Selangor, Malaysia; gs54608@student.upm.edu.my (Y.S.L.); intandiana@upm.edu.my (I.D.M.A.); 3 Centre for Fundamental and Frontier Sciences in Nanostructure Self-Assembly, Department of Chemistry, Faculty of Science, Universiti Malaya, Kuala Lumpur 50603, Selangor, Malaysia; nooridayu@um.edu.my; 4 Masonic Medical Research Institute, 2150 Bleecker St, Utica, NY 13501, USA; jcb.bhuvana@gmail.com; 5 Department of Pharmaceutical Technology, School of Pharmacy, International Medical University, Jalan Jalil Perkasa 19, Bukit Jalil, Kuala Lumpur 57000, Selangor, Malaysia

**Keywords:** liquid crystalline nanoparticles (LCNPs), cubosomes, hexosomes, drug delivery systems, vaccine delivery, theranostics

## Abstract

Due to their distinctive structural features, lyotropic nonlamellar liquid crystalline nanoparticles (LCNPs), such as cubosomes and hexosomes, are considered effective drug delivery systems. Cubosomes have a lipid bilayer that makes a membrane lattice with two water channels that are intertwined. Hexosomes are inverse hexagonal phases made of an infinite number of hexagonal lattices that are tightly connected with water channels. These nanostructures are often stabilized by surfactants. The structure’s membrane has a much larger surface area than that of other lipid nanoparticles, which makes it possible to load therapeutic molecules. In addition, the composition of mesophases can be modified by pore diameters, thus influencing drug release. Much research has been conducted in recent years to improve their preparation and characterization, as well as to control drug release and improve the efficacy of loaded bioactive chemicals. This article reviews current advances in LCNP technology that permit their application, as well as design ideas for revolutionary biomedical applications. Furthermore, we have provided a summary of the application of LCNPs based on the administration routes, including the pharmacokinetic modulation property.

## 1. Introduction

The application of nanotechnology in the field of medicine, also known as “nanomedicine”, has been one of the popular topics for various research studies to develop new and advanced drug substances or products in the pharmaceutical market. It employs engineered nanosized particulate systems ranging from 1 to 100 nm for the diagnosis, monitoring, prevention, and treatment of diseases, on top of the efficient delivery of bioactive molecules [1]. As the membranes of the cells are made up of proteins and lipids that congregate, they are highly curved in non-lamellar phases. These are crucial for cellular functions including cytokinetic abscission, filopodial extension, and membrane trafficking [2,3,4]. The rising interest over the recent decade in biomimetic nanocarrier systems that mimic the curvature of cellular membranes has been due to the remarkable advantages portrayed by membrane curvature. This includes a greater membrane surface area to volume ratio, better hydrophobic and membrane protein-loading capacities, as well as membrane stress variability when compared to planar structures [3]. Among the variety of lipid-based nano-self-assemblies that have been extensively studied for their application in drug and vaccine delivery, disease treatment and diagnosis, cosmetics, and biosensing purposes, liquid crystalline (LC) phases such as lyotropic liquid crystalline lipid nanoparticles (LCNPs) have been a promising class and an efficient tool for the previously mentioned purposes that have received great attention. Currently, LCNPs are being widely explored in numerous research studies [2,5].

The LC phase is a state of matter that exists between liquid and solid crystalline states, also known as mesophases. A compound exhibiting the LC phase is termed a mesogen. LC compounds are conventionally classified into two categories: thermotropic (phase transition according to a change in temperature) and lyotropic (phase transition due to both temperature and the concentration of the mesogen in the solvent) [6]. As of now, thermotropic LC compounds are mostly used to make materials for displays and to store information [7]. Besides display materials, thermotropic LC compounds are also used as information storage materials, optical couplers, optical waveguides, and sensors for chemical and biological analytes [8]. The highly versatile lyotropic LC compound, which is based on the amphiphilic nature of the constituent molecules, is widely used in the production of many consumer products as well as drug delivery systems [9]. The combination of lyotropic LC compounds and nanotechnology has made it possible to make LCNPs, which are new carriers that can deliver drugs through the oral route. In several examples, the incorporation of biocompatible, non-toxic excipients in the LC structure can protect pH-sensitive active ingredients from being degraded by harsh gastrointestinal conditions [10]. Sustained drug release has also been demonstrated for LCNPs, which reduces drug toxicity. Moreover, targeted drug delivery, enhanced drug bioavailability, drug stabilization, an improved pharmacokinetic profile, and a prolonged residence time can be achieved [3].

**Figure 1:**
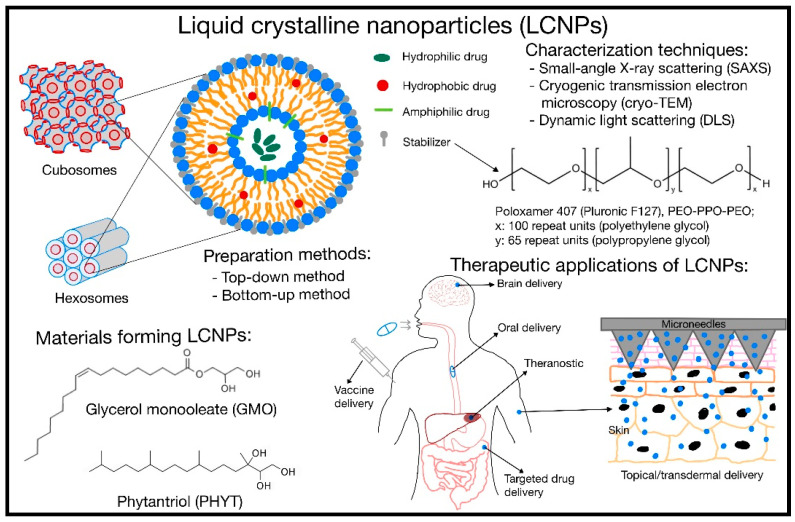
A summary of the common lipids and preparation methods used to make liquid crystalline nanoparticles (LCNPs), as well as their possible characterization techniques and therapeutic potentials.

The first part of this review mainly provides a brief overview of commonly studied lipids forming non-lamellar mesophases, preparation methods for LCNPs, as well as the use of stabilizers in stabilizing LCNPs. Next, we summarize the various characterization techniques to evaluate the potential of LCNPs in drug delivery systems. Lastly, we highlight selected therapeutic applications such as oral, topical, vaccine, targeted, theranostics, and brain drug delivery. The summary is sketched in Figure 1 and examples of therapeutics applications are presented Table 1. 

## 2. Materials, Preparation Method, and Stabilizers

**Figure 2:**
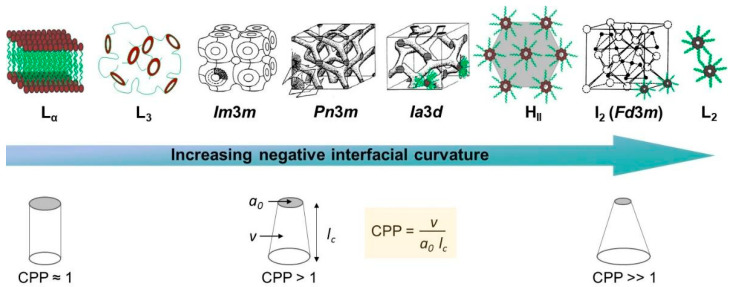
Schematic of self-assembly structures of commonly observed lyotropic liquid crystalline phases corresponding to critical packing parameter (CPP) values. This parameter is given as $CPP=v/a_{0}l_{c}$ where v is the hydrophobic chain volume, $a_{0}$ is the effective headgroup area, and $l_{c}$ is the effective hydrophobic chain length. For CPP≈1, lamellar L_α_ phase is observed with zero mean curvature. In order of increasing negative curvature (CPP>1), these are sponge L_3_, inverse bicontinuous cubic *Im*3*m*, *Pn*3*m*, *Ia*3*d*, hexagonal H_II_, inverse discontinuous micellar cubic *Fd*3*m*, and inverse micelles L_2_. Reproduced in part from [W.F.N. Wan Iskandar et al., Colloids and Surfaces A: Physicochemical and Engineering Aspects 623 (2021) 126697] [50] with permission from Elsevier.

The self-assembly of lipid molecules in an aqueous environment into lamellar and non-lamellar (bicontinuous cubic and hexagonal) phases has both attracted fundamental research interest and been of use to various applications in the medicinal field. Depending on the lipid structure and the external conditions, a wide variety of nanostructures can be formed. The lipid interfacial curvature is controlled by the molecular shape of the lipid, described by the critical packing parameter (CPP) [45]. This parameter is given as $CPP=v/a_{0}l_{c}$ where v is the volume of the hydrophobic chain, $a_{0}$ is the effective headgroup area, and $l_{c}$ is the effective hydrophobic chain length. Normal or Type I structures are anticipated when CPP<1, and if CPP≈1, a lamellar (L_α_) phase will result. On the other hand, CPP>1 will give inverse or Type II structures. In this review, the discussion has been limited to inverse phase structures as they are generally more stable in excess water and retain their structures when diluted either in bulk form or as dispersed particles [46]. Figure 2 shows the sequence of structures for inverse phases that may be found to occur with an increasing negative interfacial curvature. The L_α_ phase has zero curvature. As the negative curvature increases, a disordered bicontinuous phase known as the “swollen sponge” (L_3_) phase could form before it fully turns into the inverse bicontinuous cubic (V_II_) phase. These highly ordered bicontinuous cubic phases can be further divided into three different space group symmetries (in order of increasing negative curvature), i.e., *Im*3*m* (the primitive, P), *Pn*3*m* (the Schwarz diamond, D), and *Ia*3*d* (the Schoen gyroid, G). The phases with higher negative curvature are the inverse hexagonal (H_II_) phase and the inverse discontinuous micellar cubic (I_2_) phase with the *Fd*3*m* space group. A disordered fluid of inverse micelles (L_2_) can also be formed. Of these inverse non-lamellar liquid crystalline phases, the aqueous dispersions of the cubic phase (cubosomes) and hexagonal phase (hexosomes) are promising carriers in the drug delivery field due to their larger membrane surface area, which allows them to retain significant amounts of drugs compared to their lamellar counterparts such as liposomes [47]. Due to the unique structures that are made, pharmaceutical active ingredients with different molecular weights and polarities can be released in a controlled way and even in different amounts. In addition, cubosomes exhibit in vitro stability and aid in the ultimate in vivo dissolution upon lipolysis. Moreover, the biocompatibility and biodegradability characteristics of lipids lead to minimum toxicity despite their bioadhesive property, and they are thus allowed to be used via various administration routes [48]. The lipid self-assembly as well as the structure and stability of the phases formed can be affected by several physicochemical changes such as lipid saturation, composition, surfactant concentration, water content, pH, and temperature [49]. 

Among the numerous lipids, glycerol monooleate (GMO) and phytantriol (PHYT) are more widely utilized to form LC phases due to their biocompatibility and have been approved for in vivo use [51]. GMO (chemical name: 2,3-dihydroxypropyl oleate), also known as monoolein (MO) or Rylo MG 19, forms several mesophases upon different physicochemical parameters. Since it is non-toxic, biocompatible, and biodegradable, it is used as an emulsifier in both the food and drug industries. PHYT (chemical name: 3,7,11,15-tetramethylhexadecane-1,2,3-triol), an appropriate alternative to GMO for cubosome formation, is extensively employed in the cosmeceutical industry as a cosmetic active ingredient due to its safety, biocompatibility, and excellent mucoadhesive properties, which improve skin penetration as well as moisture retention. Similarly, the phase formation is determined by certain physicochemical factors, but PHYT has higher chemical stability (due to the phytanyl functional groups and lack of ester groups) and is thus able to preserve the LC structure against lipase activity, as opposed to GMO [52]. In addition, PHYT cubosomes possess a remarkable capacity for the sustained release of hydrophilic drug molecules, as reviewed by Rizwan et al. [53].

**Figure 3:**
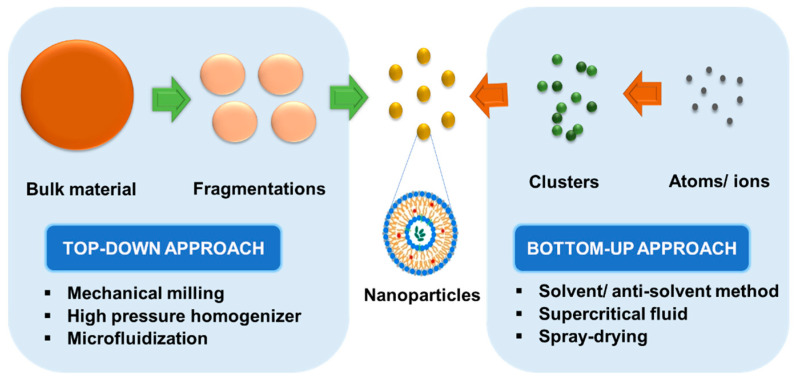
Schematic illustration of various preparation methods.

LCNP preparation strategies can be further categorized into the top-down method, where high energy from high-pressure homogenization or sonication is applied, or the bottom-up method, which involves the dilution of an isotropic solution using hydrotropes as an input factor to lower the energy input (Figure 3) [54]. The top-down approach is more attractive and has wider industrial applications than the bottom-up approach due to its ability to break down larger particle of LCNPs into a desirable size (<200 nm) and in the absence of residual solvent that can cause the physical and chemical instability of LCNPs. In addition, several techniques such as high-pressure homogenization, sonication, spontaneous emulsification, shearing, spray drying, hydrotrope incorporation, and less commonly, mechanical stirring, would form LCNPs with smaller and more uniform particle sizes. As the residual solvent could increase cellular toxicity and modify the phase behavior, high-pressure homogenization and sonication are the most frequently applied techniques to produce stable and reproducible LCNPs where a solvent is not required during the process. Another option to address the concerns includes the use of alternative solvents such as polyglycerol ester or propylene glycol. Furthermore, vesicles could be formed as a by-product in sonicated dispersion, but this can be solved by heat cycling [3]. Under certain conditions where the aqueous dispersion of LCNPs is unstable, a spray-drying technique can be applied to overcome the limitation. They produce powdered cubosomes or an intermediate, which on hydration with an aqueous solvent, will reform into LCNPs [55,56].

Additionally, stabilizing agents are needed to sustain the colloidal stability of LCNPs in an aqueous environment by forming a steric or electrostatic barrier between the particles as well as to prevent the cubosome’s internal phase structure from being disrupted. They reside on the surface of cubosomes to prevent flocculation and ensure the stability of dispersions, which eventually contribute to the compound’s stability and lipid mixture phase morphology [57]. Pluronic F127, also known as Poloxamer 407, is a non-ionic triblock copolymer (PEO-PPO-PEO) composed of polyethylene glycol (PEO) and polypropylene glycol (PPO). It has been the most widely used stabilizing agent in several lipid systems, including GMO and PHYT. GMO dispersions stabilized with low concentrations of Pluronic F127 produce cubosomes with *Pn3m* internal geometry, but the overall quality is relatively poor due to the presence of visual aggregates. On the contrary, higher Pluronic F127 concentrations used in GMO dispersion stabilization form cubosomes with an *Im3m* internal structure in which the dispersions are aggregate-free. Furthermore, *Pn*3*m* cubosomes are formed in high-Pluronic-F127 stabilized phytantriol dispersions. This clearly shows that LCNPs’ steric stabilization is subject to the concentration of the stabilizer in addition to the stabilizer structure and PEO chain length. Nonetheless, other stabilizers, including Pluronic 108, Tetronic 908, Tween 80, and Myrj 59 show better stabilizing effects than Pluronic 127, with Pluronic 108 and Tetronic 908 showing the best stabilizing effects [49,58].

## 3. Characterization Techniques

**Figure 4:**
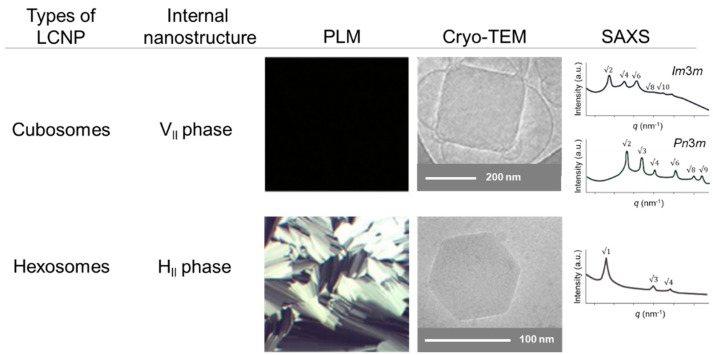
Representative PLM textures, cryo-TEM images, and SAXS patterns of lipid nanoparticles for cubosomes and hexosomes. PLM: polarized light microscopy; cryo-TEM: cryo-transmission electron microscopy; SAXS: small-angle X-ray scattering. Adapted with permission from [66,67].

The use of LCNPs for drug delivery has advantages over conventional drug delivery methods, including high stability, target specificity, and the ability to deliver both hydrophobic and hydrophilic drugs. It is crucial to comprehend how the characteristics of drug carriers with nanometric dimensions affect their in vivo behavior and distribution due to their unique physicochemical properties. LCNPs can be prepared using a variety of techniques; therefore, it is essential to define their structures and properties to assess their potential as drug delivery platforms. There are numerous techniques to determine the LCNPs system’s stability, type of mesophase, shape, and internal nanostructures. Finding suitable, reliable, and robust techniques that can be employed for this reason is therefore required. The methods that are most routinely used to evaluate the structural aspects of nanocarriers are dynamic light scattering (DLS), polarized light microscopy (PLM), small-angle X-ray scattering (SAXS), and cryo-electron microscopy (cryo-TEM). These techniques can be used to determine the size, stability, shape, morphology, and dispersion state of nanometric systems [59,60]. Each of the techniques has certain strengths and restrictions in characterizing and analyzing nanoparticles, due to the inherent properties of nanoparticles such as their particle size being too small or their low quantity compared to the bulk materials; hence, a suitable technique should be chosen according to the properties that need to be investigated. Nevertheless, a combination of characterization approaches is often required [61]. Figure 4 illustrates the characteristic PLM, cryo-TEM, and SAXS pictures of cubosomes and hexosomes. No attempt has been made thus far to provide a comprehensive review encompassing the most-known characterization methodologies for LCNPs. For the benefit of the general reader, we have henceforth outlined the function, underlying concept, and limitations of several of the most common characterization techniques. Existing reviews have introduced and explored in detail the characterization technique for LCNPs.

DLS provides information on the particle size and its distribution as well as the agglomeration state of nanoparticles, which determines the stability of dispersions. This technique is based on the temporal fluctuations of the elastic scattering intensity of light, i.e., Rayleigh scattering, caused by the Brownian diffusion of spherical particles, where the Brownian movement of the particles is related to an equivalent hydrodynamic diameter using the Stokes–Einstein relationship. Alongside particle size, this light scattering system may also determine the molecular weight and the surface charge (zeta potential). One of the drawbacks of DLS is that nanomaterials tend to aggregate in water, changing their size and surface properties. As a result, the size may be overestimated and the size distribution may be altered due to environmental dependence [59,60]. 

PLM is commonly used for the preliminary phase identification of lyotropic liquid crystals and offers the easiest way of characterizing the phase properties of lipid–water systems. When using an additional λ-plate, anisotropic systems cause a deviation in the plane of polarized light (birefringence, similar to real crystals), resulting in typical black and white images, or colored textures [61]. The technique could distinguish between classic mesophases based on their characteristic textures. The birefringence texture is a characteristic of the lamellar and hexagonal phases, in which the former typically show oily streaks with “Maltese crosses”, while the latter can be recognized by a fan-like texture. The cubic phase does not show any texture due to its isotropic nature [59]. However, the light intensity is based on the optic axis angle of the observed material relative to the transmission axes of the polarization filters. In other words, PLM is not a quantitative technique, but rather qualitative [62]. However, it is limited to particle dimensions in the micron or submicron range.

Cryo-TEM is used for visualization of the morphological characteristics and to determine the size of nanoparticles. Moreover, cryo-TEM can also be used to determine the mesophase using fast Fourier transform analysis [63]. In principle, this technique uses powerful electron beams, thereby providing a higher resolution and better detail. The morphology of the sample is preserved upon careful freezing (cryo) [60]. Since cryo-TEM involves a high-resolution imaging technique, it is only possible to view a minute part of the sample, resulting in poor statistical sampling [61]. Cryo-field emission scanning electron microscopy (cryo-FESEM) is another alternative technique to study the structure of nanoparticles. It provides more superficial information regarding the materials than cryo-TEM but is not able to determine the type of mesophase [63]. 

The SAXS technique is a non-invasive technique, which allows elastic scattering of incident X-ray in very small angles (usually <1°) to determine the nanochannel size of LC particles. In addition, it provides detailed structural analysis and physical information of the sample including the dimensions, lattice type, and surface-to-volume ratio, with the size ranging from 1 to 100 nm [61]. Moreover, the absolute position of the Bragg peaks enables the calculation of the average lattice parameter, a, of the matrices, from the corresponding interplanar spacing, d (d=2π/q, where q refers to the scattering vector), using the proper scattering law for the phase structure [64]. Comparing the obtained Bragg peaks with databases allows the determination of the internal structure of nanoparticles since every phase has a typical peak ratio. For the three different space group symmetries of bicontinuous cubic phases, i.e., *Im*3*m*, *Pn*3*m*, and *Ia*3*d*, their first five structure peak ratios are: $\sqrt{2},\sqrt{4},\sqrt{6},\sqrt{8},\sqrt{10}$; $\sqrt{2},\sqrt{3},\sqrt{4},\sqrt{6},\sqrt{8}$, and $\sqrt{6},\sqrt{8},\sqrt{14},\sqrt{16},\sqrt{20}$, respectively. For the hexagonal phase, their first five structure peak ratios are: $\sqrt{1},\sqrt{3},\sqrt{4},\sqrt{7},\mathrm{and}\sqrt{9}$. For the lamellar phase, it exhibited equidistant peaks. Due to the fact that LCNPs are made with low-electron-density atoms (i.e., H, C, N, and O) and laboratory X-ray sources only provide a small X-ray flux, data acquisition is slow and of limited quality using a standard laboratory SAXS instrument. A synchrotron X-ray source is preferred for higher resolution measurements and shorter acquisition times [63]. In most cases, SAXS data will be supported by wide-angle X-ray scattering (WAXS) or X-ray diffraction (XRD). They are similar to SAXS, but because the detector and sample are closer together, they can observe scattering/diffraction maxima at larger angles. Hence, they are sensitive to the size of the short-range order region, which is suitable for the crystallite size [61]. Frost et al. conducted a study to evaluate the crystalline fraction of high-amylose thermoplastic starch used to produce films. Changes in the starch films, which included full or partial gelatinization, retrogradation, and crystallinity, were determined by WAXS [65]. Another technique that can be used for particle size measurement includes small-angle neutron scattering (SANS) which uses elastic neutron scattering to determine the polymer layer static structure. It is applicable to nanoparticles of a few nm to 1 mm. Nonetheless, SANS outperforms SAXS in terms of its benefits such as strong magnetic moments scattering and a higher sensitivity to light elements, as well as the ability to label isotopes, but SANS is not applicable for thin films and substrates [59,61]. 

## 4. Pharmacokinetic Modulation Using LCNPs

Significant advances in research on the pharmacokinetics of nanopharmaceuticals have been evident over the past few decades, including in silico physiologically based pharmacokinetic modelling (PBPK), computational quantitative structure–activity relationship (QSAR) approaches, and in vivo pharmacokinetic studies in animal models. These have important implications in the evaluation of the absorption, distribution, metabolism, and excretion of NPs to decipher the in vivo fate of NPs. Research over the years has shown that LCNPs can offer multifunctional roles with regard to biocompatibility, in vivo bioavailability, and targeted distribution to the site of disease. Interestingly, in vivo phase transitions of LCNPs have been investigated in several studies. For example, Pham et al. reported that gastroretentive phytantriol and tributyrin-based cubosomes were formed because of digestion in an in vivo rat model [68]. Using micro-X-ray computed tomography (CT)-imaging of gold (Au), integrated cubic-phase NPs improved the absorption kinetics of the cubosomal formulation of a model encapsulated drug, cinnarizine, which contained both phytantriol and tributyrin in an optimized compositional ratio. In situ generation of non-lamellar inverse bicontinuous cubic LCNPs in synovial fluid was also revealed as a potential injectable depot formulation for intra-articular treatment [69]. Strategies for the evasion of clearance mechanisms by long-circulating LCNPs have therefore included the modulation of size, shape and topology, surface charge, interfacial properties, and PEGylation [70,71]. Fusogenic properties [72], attenuation of P-gp efflux [73], endosomal escape [74], and modulation of interactions with lymph node macrophages have been effectively demonstrated for LCNPs [75]. Furthermore, LCNPs have been engineered to be stimuli-responsive and provide a ligand-targeted therapeutic action at the site of disease [76,77,78,79]. Modulation of the controlled release of a model hydrophobic substance (clofazimine salt) [80], and an anticancer agent (PTX) [81] was shown by lipid cubic phases adjuvanted with a lipase inhibitor, i.e., tetrahydrolipstatin. Taken together, these modified structures of LCNPs are capable of bypassing rapid systemic elimination and contributing to improved pharmacokinetic parameters.

Notably, in animal models, improved pharmacokinetics were exhibited for the oral delivery of a range of therapeutic molecules, including celastrol [82], tamoxifen [83], telmisartan [84], rosuvastatin [85], and *p*-amino benzoic acid [86]. In the study by Yasser et al., a compound-based oral tablet formulation was developed for telmisartan, in which the pharmacokinetics examined in male albino rabbits proved increased bioavailability and superior pharmacokinetics in comparison with a commercial telmisartan tablet for hypertension treatment [84]. Moreover, Jeon et al. investigated omega-3 ethyl ester compound lipids and oleic-acid-based LCNPs, which retained high in vitro dissolution rates in simulated gastrointestinal tract (GIT) conditions [87]. Furthermore, oral bioavailability in male beagle dogs was superior to that of marketed Omacor soft capsules containing purified omega-3 polyunsaturated fatty acids. Concerning subcutaneously injected LCNPs, Shiadeh et al. reported the pharmacokinetic assessment of risperidone-loaded LCNPs in rabbits [88,89]. Both of the in-situ-forming gel formulations that consisted of glycerol dioleate (GDO)–phosphatidylcholine (PC) and PC in combination with sorbitol monooleate (PC:SMO), Tween grade 80, and tocopherol acetate (TA) were optimized and compared with Risperdal CONSTA^®^ (Janssen Pharmaceuticals, NJ, USA). On the other hand, rat models have been employed for the pharmacokinetic evaluation of (i) 5-fluorouracil-loaded Pluronic F127-MO cubosomes that have additionally shown targeted accumulation in liver tissues [90], (ii) a leuprolide-acetate-loaded hexagonal LC matrix composed of PC, SMO, and tocopherol acetate (TA) [91], (iii) a PC:SMO LC system for the subcutaneous delivery of naltrexone [92], and (iv) technetium-99 m (99mTc)-radiolabeled phytantriol and oleic acid-based hexosomes [75]. Interestingly, the utilization of single-photon emission computed tomography (SPECT) in combination with CT was described for monitoring the in vivo biodistribution of the 99mTc-radiolabeled hexosomes. Pharmacokinetic studies of LCNPs for direct application via the ocular or otic routes have been carried out for vancomycin-loaded cubosomes [93], chitosan-coated voriconazole-loaded cubosomes [94], and nerve-growth-factor-loaded cubosomes [95], whereas LCNPs encapsulating luliconazole [96], diclofenac [97], and tofacitinib [98,99] have been examined in terms of the dermatokinetics associated with transdermal application. Further, in vivo pharmacokinetic and biodistribution modeling of brain-targeted delivery by LCNPs has been demonstrated with an intranasal in situ hexosomal gel carrier of vinpocetine [100] and RhoB-incorporated cubosomes, which facilitated greater drug availability in the plasma and brain homogenates of rats, and a higher NP uptake in the brain of zebrafish larvae, respectively. In particular, the non-invasive administration of nanomedicines with enhanced pharmacokinetics garners clinical relevance as they can potentially lead to greater patient compliance and therapeutic efficacy. At the same time, further advances can be expected for pharmacokinetic modulation using LCNPs. Partly, this may involve functionalization of LCNPs to potentiate higher penetration across physicochemical barriers in the respective routes of administration [101,102,103].

## 5. Therapeutics Application

### 5.1. Oral Delivery

Oral drug delivery is the most desirable and preferred administration pathway among other routes of drug delivery such as the transdermal route, intranasal route, and parenteral route. Oral administration is normally preserved to be utilized in treating long-term diseases rather than acute conditions, which require immediate action, as oral drugs require some time to exert their therapeutic effects. In fact, patients demonstrate higher compliance with drugs delivered via the oral route due to its non-invasiveness and convenience of administration [104]. However, some factors will affect the efficacy of oral delivery, such as the pH of gastric fluid, gastrointestinal motility, permeability of drugs across biological membranes, and hepatic first-pass metabolism, which, result in low bioavailability and thereby lead to poor therapeutic outcomes [104,105]. Many studies and reviews have recently been published on the advanced development of nanoparticles such as cubosomes for oral drug delivery applications [13,15,16,17,104,105,106,107,108]. These nanoparticles have been suggested as a promising carrier for oral delivery for loading, entrapping, and encapsulating the drugs with hydrophilic, lipophilic, or amphiphilic properties due to the distinctive characteristics and structures of nanoparticles [105,106,108]. They have provided a lot of advantages in pharmaceutical oral formulations such as sustaining and controlling the drug release, enhancing the bioavailability of drugs, improving the permeability of drugs across biological membranes, and improving the efficacy and delivery of oral drugs [106]. 

One example comprises cubosomal NPs, which have been used to improve the bioavailability of cyclosporine A and simvastatin. Cyclosporin A has poor permeability and poor water solubility, whereas simvastatin has poor water solubility, although its permeability is high [109]. As cubosomes are lyotropic, they can encapsulate poorly water-soluble drugs in their lipid bilayers and render them soluble [105]. The encapsulation can also protect the drug payload from unfavorable physiological environments, such as extreme pH and temperature, besides protecting the drugs from enzymatic biodegradation [104]. The encapsulation technique is also applied to another example, a potential anticancer drug, curcumin. Curcumin has low solubility in water and low bioavailability as it is rapidly metabolized by the liver. Chang et al. demonstrated that curcumin-loaded cubosomes have a greater effectiveness against a murine melanoma cell-line as stronger cytotoxic activities are performed by the curcumin-loaded cubosomes [13]. Moreover, the muco-bioadhesiveness of nanoparticles also plays an important role oral drug delivery [105,106,107]. Nanoparticles, such as GMO cubosomes, enhance drug permeation across the membrane by exerting fusogenic properties and thereby increasing bioavailability. Besides enhancing drug permeation, the drug-loaded cubosomes also exert sustained-released properties due to their strong adsorption to the epithelium [107]. 

Furthermore, pH-sensitive lyotropic cubosomes have tremendous advantages in oral chemotherapy drug delivery systems. The pH of the environment in normal cells is slightly alkaline, which is usually approximately 7.4. However, the surroundings of tumor cells are usually acidic because tumor cells produce lactic acid as metabolic waste during the rapid development of cancer cells. Due to the pH differences, the anticancer-drug-loaded cubosomes will have a higher selectivity towards cancer cells; hence, the accumulation of these cubosomes will also be higher in tumor cells compared to normal cells [107]. One study revealed the effects of cubosomes on animals, particularly in treated rats, where the antitumor efficacy and drug bioavailability improved when doxorubicin was loaded in phytantriol cubosomes. The risk of cardiotoxicity was also reduced when compared to intravenously administered Adriamycin^®^. The enhanced permeation and retention (EPR) effect provided by the cubosomes has led to a higher concentration of drug-loaded nanoparticles at the tumor sites, whereas the longer circulation half-life has enhanced oral drug delivery [106]. 

In addition, the use of nanoparticles can enable oral drugs to be delivered to the target sites, and hence, the poor oral delivery can be resolved. For example, cubosomes have reportedly brought advantages to coenzyme Q_10_ (CoQ_10_) in terms of its poor oral delivery and hepatoprotective activity. Since CoQ_10_ has a high molecular weight and low solubility, it will encounter some unwanted limitations such as poor bioavailability during administration. However, this is no longer a concern as cubosomes can encapsulate CoQ_10_ in the lipid bilayers and improve its delivery. In the same study, hepatoprotective effect in the thioacetamide (TAA)-induced hepatotoxicity in rats was also investigated. In vitro studies revealed that CoQ_10_-loaded cubosomal NPs can last up to 48 h. On the other hand, the in vivo study revealed that the markers of oxidative stress and liver functions were maintained within the acceptable ranges when cubosomes were used. These results demonstrated that the cubosomal nanoparticles have the potential to improve the efficacy of CoQ_10_ in terms of its hepatoprotective effect [15]. Another study reported by Yang et al. found that loading amphotericin B to GMO cubosomes significantly improved oral delivery compared to the intravenously administered clinical formulation Fungizone^®^ [16]. Moreover, Nasr et al. have reported that the use of cubosomal nanoparticles in loading the antidiabetic drug, gliclazide, has improved the therapeutic activity and oral bioavailability. The gliclazide cubosomal formulation was compared with the conventional gliclazide suspension in the study and the bioavailability in rats increased by two-fold compared to the suspension. Blood glucose levels also significantly reduced when the gliclazide-loaded cubosomes were administered to the rats [17]. 

Despite the benefits of cubosomes in pharmaceutical oral formulations, further studies are still required to provide stronger evidence for their use in oral delivery.

### 5.2. Topical/Transdermal Delivery

Topical delivery involves the drug being applied to a particular part of the body, such as the eyes, nose, and skin for localized treatment. It provides some advantages such as causing low fluctuations of the drug concentration due to its sustained and controlled drug release properties, higher patient compliance due to the non-invasiveness of this route of administration, and its highly localized drug delivery as it is topically applied on a particular part of the body. In addition, topical delivery can overcome the toxicity or systemic side effects induced by other administration routes such as the oral or injectable route [110]. 

The first topical delivery method discussed in this review is ocular delivery, where the drug is delivered to the eyes to manage eye diseases. Conventional ocular formulations have some limitations, such as low bioavailability, a low retention time, and low corneal permeability. Biological barriers of the eyes will limit the passage of drugs into the eyes and hence the therapeutic effects will be affected [111]. LCNPs can resolve the low bioavailability and low corneal permeability issues, and thus, they may be used as alternative approaches for ocular diseases such as glaucoma [112]. In one study, the assessment of ex vivo permeation showed that the unique compound structures increased the retention time of the formulations on the cornea. The in vivo examination in the same study showed that the cubosomal formulation resulted in improved effectiveness and required a lesser frequency of administration than the conventional eye drops [113]. In another study, the cubosomes were loaded with ketoconazole for ocular delivery to treat fungal infections. The results showed that cubosomal formulations improved the antifungal action and drug permeability. The minimal inhibitory concentration (MIC) values were significantly reduced when compared to the conventional ocular formulations [18]. However, the antiglaucoma drug latanoprost was loaded into cubosomes for glaucoma treatment in another study. The formulation outperformed the marketed formulations, and in vitro studies revealed that latanoprost was released continuously from the compound formulation [19]. In addition, uveitis in rabbits was effectively treated with beclomethasone-dipropionate-loaded cubosomes; hence, this can be a potential alternative formulation compared to other current approaches such as intravitreal or periocular injections [20]. Boge et al. also demonstrated that there was no skin irritation when the antimicrobial peptide LL-37 was delivered topically using cubosomes [114]. 

Aside from ocular delivery, cubosomes may be a potential carrier for drug loading to treat inner ear diseases [115]. Al-Mahallawi et al. conducted an evaluation for both the in vitro and in vivo study of the management of otitis externa in rabbits using norfloxacin-loaded cubosomes [21]. According to the histopathological result, this formulation was found to be safe to apply to rabbit’s ears. It could enhance drug permeation, and the accumulation of drugs in rabbits’ ear skin was found to be high. However, the evidence for its usage is still limited, and hence, it requires further study to prove its effectiveness. 

The usage of cubosomes in transdermal delivery is becoming popular nowadays. The transdermal route of administration provides many advantages such as by-passing first-pass metabolism as well as protecting the drugs from gastrointestinal degradation and enzymatic degradation. However, the therapeutic effects of drugs delivered by the transdermal route are reduced by the stratum corneum, which is the outermost layer of the skin. In order to reach systemic circulation, the drugs delivered by the transdermal route need to permeate the skin. However, the stratum corneum will limit the passage of drugs into the skin, and hence, only small amounts of drug molecules are able to pass through the layer [110]. With the advancement in technology, a drug for transdermal delivery can be loaded with cubosomal nanoparticles to circumvent this limitation. Several studies have shown that the typical ordered structure of cubosomes is similar to that of the stratum corneum, provided that the colloidal polymer is added or the hydrogel dilution is performed. Moreover, a targeted and sustained transdermal delivery system can be achieved by using phytantriol-based cubosomes or GMO cubosomes. For example, capsaicin has been shown to exhibit sustained skin retention and release, despite having lower percutaneous absorption than conventional formulations [116]. Another example is the phytantriol cubosomes that are used to encapsulate rapamycin, where Pluronic F127 is used to stabilize the cubosomes. The results of this study not only indicated that the efficiency after encapsulation was higher than 95%, but the in vitro drug release profile also showed that rapamycin had sustained-release properties after cubosomes were incorporated [23]. Therefore, cubosomes might be a potential candidate for developing transdermal formulations to increase therapeutic outcomes in the treatment of skin diseases. In another study, it was demonstrated that tenoxicam-loaded hyalcubosomes such as cubosomes, which contain sodium hyaluronate, might be safe and effective in relieving or alleviating the symptoms of osteoarthritis, after treatment for 8 weeks [24]. The effectiveness in terms of permeation or penetration across the skin and retention properties were also assessed in the same study. In addition, transdermal delivery can be an alternative to oral delivery when oral administration is not feasible. Colchicine used in the treatment of gout will produce side effects if it is administered via the oral route. Nasr et al. demonstrated that colchicine-loaded cubosomal nanoparticles can reduce the associated side effects [25]. In a review, it was concluded that nanoparticles loaded with dapsone increased the drug solubilization, resulted in better control of drug release, and enabled targeting [26]. The drug absorption was significantly increased when colchicine-loaded cubosomes were used compared with the oral formulations. Nithya et al. have also shown the permeation of dapsone through the skin is enhanced when it is incorporated with cubosomal nanoparticles [26].

### 5.3. Brain Delivery

Drug delivery to the brain has remained a significant challenge because of the restriction of the blood–brain barrier (BBB). The BBB is an essential diffusion barrier that protect the normal functions of the brain by impeding the transition of most compounds from the blood to the brain. In contrast, the BBB of patients with neurodegenerative disorders such as Parkinson’s disease and Alzheimer’s disease (AD) is disrupted due to the remodeling of protein complexes in inter-endothelial junctions [117]. Drug delivery to the brain mostly depends on the structure and lipophilicity of the molecules. The BBB is impermeable to most macromolecules, with the exception of small molecules that are lipid soluble and have a molecular weight of less than 400 Da [117,118]. Since the population of patients with central nervous system (CNS) disorders are increasing, global drug development must grow rapidly [117]. However, the development of CNS drugs has the poorest success rate compared with that of non-CNS drugs. However, a longer development time is required for CNS drugs. A recent study has reported that the development of CNS drugs becomes challenging due to the complexity of the brain, side effects, and the impermeable BBB [107]. Some BBB delivery technologies have been found to allow the entry of drugs into the brain from the blood such as cerebrospinal fluid (CSF) injection [119]. Nevertheless, CSF injection shows limited drug penetration into the parenchyma of the brain. Zhou et al. stated that nanocarriers have been investigated to find a mechanism that could aid in the delivery of CNS medicines [120]. There are different types of nanocarriers, such as liposomes, ethosomes, SLN, and cubosomes. The use of nanocarriers to deliver CNS drugs may aid in navigating the biological interface and change the treatment of a variety of life-threatening brain illnesses. Cubosomes have a larger lipid compartment ratio in their structure, which permits them to hold large numbers of ‘fatty’ molecules. Thus, cubosomes are particularly beneficial for delivering lipophilic drug molecules to the brain because many tiny lipophilic drug molecules have adequate BBB permeability. However, most of the lipophilic small-molecule drug are frequently the substrates of drug efflux transporters such as P-glycoprotein (P-gp) which is widely expressed on capillary endothelial cells. In addition, cubosomes have fusogenic capabilities with cellular membranes and might potentially open new avenues to distribute medications through the BBB [28]. 

Nanocarriers may increase the drug concentration at the BBB surface and provide a better chance of it crossing the BBB than conventional formulations by lengthening the drug circulation period in the blood. One of the examples is using cubosomes and hexosomes to deliver phenytoin to the brain. Phenytoin is the primary treatment of status epilepticus and is normally administered intravenously. However, the intravenous route is not the most suitable for phenytoin due to its poor water solubility and complications such as severe pain, tissue necrosis, and edema. The brain and plasma concentrations of phenytoin are significantly higher after being administered in cubosomes and hexosomes. This shows that the use of cubosomes and hexosomes is useful to enhance BBB penetration and transport of CNS drugs [27]. Furthermore, Azhari et al. investigated cubosomes produced with Tween 80, Pluronic F127, and Pluronic F68 surfactants (previously proven to enable nanocarriers to target the BBB), in a zebrafish model, whereby the uptake of lissamine rhodamine (RhoB), i.e., a model therapeutic molecule with low BBB permeability, was significantly increased [28]. In an in vivo study, Gelperina et al. found that doxorubicin and loperamide were delivered more effectively to the brain when given in PLGA nanoparticles or poly (lactide-co-glycolide) encapsulated with Pluronic F68 [29]. However, the intranasal route is one of the effective routes that can be used to deliver drugs into brain [112,121]. In this method, drugs can bypass the BBB and reach the brain directly. Wu et al. discovered the efficacy of pegylated cubosomes, which consist of Pluronic 127, maleimide–PEG–oleate, and GMO, functionalized with an odorranalectin for the nose-to-brain delivery of the S14G-HN peptide, a current peptide that acts against cerebral ischemia and AD. The study reported that cubosomes enhanced the neuroprotective effect of the S14G-HN peptide [30]. Moreover, Nguyen reported that cubosomes with Pluronic F127 can adsorb to the plasma membrane easily but may cause destabilization. In contrast, GMO cubosomes are less likely to contact and disrupt the membrane directly due to steric stabilization. These findings conclude that liquid crystalline nanoparticles such as cubosomes are ideal drug transporters and less toxic. The author also suggests that nanoparticles can cross via receptor-mediated transcytosis, rather than interacting with the cell membrane directly for uptake [121]. Hence, cubosomes provide several benefits for brain targeting, such as controlled drug release, high entrapment efficiency, thermodynamic stability, biocompatibility, and their bioadhesive properties.

### 5.4. Targeted Drug Delivery

Targeted drug delivery, also known as smart drug delivery, is a method of giving medication to a patient, for which the concentration of the medication is greater in a specific part of the body. Targeted drug delivery is largely found in nanomedicine, which intends to use nanoparticle-mediated medication delivery to overcome the drawbacks of traditional drug delivery. These nanoparticles would be drug-loaded and targeted to specific areas of the body with diseased tissues only, avoiding interaction with healthy tissues. The goal of this delivery system is to extend, localize, target, and have therapeutic contact with pathogenic tissues, making it safe. The standard drug delivery mechanism is drug absorption across a biological membrane, while a targeted release system administers the medication in a dosage form. The benefits of the targeted release system include a reduction in the frequency of a patient’s medications as well as a more precise dosage. Liquid-based hexosomes and cubosomes are nanoparticles that contain an inverse bicontinuous cubic phase, non-lamellar lyotropic liquid crystalline mesophases, and an inverse hexagonal phase. A recent study reported that the values of non-lamellar LCNPs include the capacity to encapsulate hydrophilic and hydrophobic pharmaceuticals with high encapsulation efficiency, the tunability and responsiveness to external stimuli for drug release control, and the ability to protect and deliver big biomolecules such as proteins, peptides, and DNAs. In in vivo preclinical models, the efficacy of encapsulated pharmaceuticals within hexosomes and cubosomes has been shown to be improved [122]. 

Cubosomes with a porous 3D nanostructure can load hydrophobic small molecules and high-molecular-weight substances in bicontinuous cubic LC phases. Low and high-water contents in GMO cubosomes can result in cubic forms with diamond and gyroid surfaces, respectively. Pluronic F127 and 1,2-distearoyl-sn-glycero-3-phosphoethanolamine-*N*-[maleimide (PEG)] polymers were used to stabilize GMO cubosomes and encapsulate paclitaxel (PTX) [123]. When compared to unloaded PTX, this formulation resulted in a 50% reduction in tumor size [124]. Functional materials and ligands actively target cubosomes, which is a clever method for lowering the formulation toxicity in physiological settings. A biotin-based block copolymer was employed to enhance cubosomes encapsulating PTX because the cancer cells that overexpress biotin-specific receptors were actively targeted. When compared to normal cells, epithelial growth factor receptors (EGFRs) are highly expressed in ovarian cancer cells. In this way, the surface functionalization of PEGylated cubosomes containing GMO with EGFR antibody fragments resulted in the efficient in vivo targeting of aggressive ovarian cancer cells [125]. Cubosomes carrying a PTX dose of 5 mg/kg body weight in mice resulted in a 50% reduction in tumor growth in this study. This reduction could be due to the cubosomes encapsulated with EGFR 528 monoclonal antibodies properly targeting the ovarian cancer cells. 

One example of the use of cubosomes in targeted drug delivery is GMO cubosomes, which are used as nanocarriers to deliver antimicrobial peptides. Antibiotic peptides (AMPs) have exhibited a promising, viable alternative to conventional antibiotics in fighting against antibiotic resistance. However, AMPs are difficult to translate into useful pharmaceuticals naturally due to their low bioavailability and biophysical stability. Besides that, AMPs must first pass through the lipopolysaccharide-coated outer membrane (LPS) to kill Gram-negative bacteria by damaging their core membrane. Hong et al. investigated the use of liquid nanoparticles in AMP delivery and found that AMP LL-37 encapsulated in GMO liquid nanoparticles were responsive antimicrobial nanocarriers [31]. This is because nanoparticles are used to enhance the penetration of LPS layers and destroy the bacterial membrane. Hydrophilic polysaccharide chains of LPS would contribute to the interior cubic structure swelling. The LPS-induced nanostructural changes could be used for LPS-triggered AMP/drug delivery because the swelling of the LC phase has been proven to improve the rate of released encapsulated molecules. 

Over the years, lyotropic LCNPs have been intensively investigated as a beneficial nanocarrier for antitumor agents. Drugs of any kind can be incorporated due to the presence of amphiphilic channels. Freag et al. created rapamycin-loaded cubosomes to enhance the active cancer targeting and water solubility of the drug [126]. When compared to the free drug, cubosomal rapamycin showed greater cytotoxicity with a 3.35-fold improvement in bioavailability. Moreover, fluoxetine hydrochloride (FH)-loaded hexosomes enhanced in vitro drug release, improved cellular internalization, and increased cytotoxicity in a time-dependent manner [34]. In addition, Li et al. effectively formulated pH-sensitive liquid crystalline nanoparticles loaded with Brucea javanica oil and doxorubicin. In this study, the authors used a pH-induced phase change from an inverted hexagonal liquid crystalline structure (pH = 7.4, simulated normal cell environment) to cubic network architectures of open channels (pH 6.8, simulated weakly acidic pH environment around cancer cells) to efficiently deliver Brucea javanica oil and doxorubicin with enhanced antitumor activity against breast cancer cells [127].

Saber et al. developed nanocubosomes as nanocarriers for anticancer therapies. The nanocubosomes containing cisplatin and cisplatin–metformin combinations were tested on HCT-116 cells. The authors discovered that, when compared to unformulated cisplatin, the nanocubosomal formulations had a better cytotoxic impact. The cytotoxic effect was amplified when metformin, an indirect mTOR inhibitor, was added to cisplatin nanocubosomes. Drug-loaded nanocubosomes caused energy and glucose levels to drop, thus activating AMPK and inhibiting mTOR. Furthermore, drug-loaded nanocubosomes caused a significant rise in ROS levels, as evidenced by an increase in NADPH oxidase, suppression of LDH, and an increase in caspase-3 [35]. In addition, LCNPs have also been used to transport albendazole (ABZ), a powerful inhibitor of severe carcinoma types, into the body. ABZ has poor bioavailability; thus, it is not suitable for administration via the oral route. Saber et al. reported that ABZ cubosomes had a two-fold-higher bioavailability, which may be due to the fact that the biological membrane and the cubosomal lipid bilayer are similar. The authors revealed that ABZ-loaded cubosomes could be a possible drug delivery system that needs further investigation for various cancers [36]. 

Moreover, Thapa et al. prepared a layer-by-layer polymer-assembled LCNPs based on GMO stabilized by Pluronic F127 for delivering sorafenib, a low solubility medication used to treat advanced hepatocellular carcinoma [37]. The coating of nanoparticles was presented to overcome some of the drawbacks of intravenous LCNP delivery, such as bioadhesion, LCNP-induced hemolysis, and rapid clearance from the blood. It also enabled the regulated release of the drug, precise administration, and increased therapeutic anticancer activity indices [128]. Multi-layered LCNPs increase superior apoptotic effects and high cellular absorption. In vivo investigations revealed that better expression of apoptotic markers and increased cytotoxicity resulted in improved antitumor efficacy with minimal adverse effects. This research sheds lights on the fact that LCNs have the potential to be used as dual medication delivery systems to treat metastatic breast cancer. Similarly, Aleandri et al. created cubosomes for PTX, a weakly soluble anticancer medication, that were stabilized by modified Pluronic F108 [128]. In this study, Pluronic F108 was combined with biotin and used to actively target overexpressed biotin receptors in HeLa cells. The data demonstrated that biotin receptor-mediated endocytosis improved the cellular absorption of PTX-loaded biotinylated cubosomes in HeLa cells. 

Cubosomes could also be used to transport a neuroprotective drug to damaged retinal ganglion cells (RGCs) caused by acute intraocular pressure (IOP) elevation in severe retinal injury. Ding et al. investigated the implication of LCNPs on the targeted drug delivery of glaucoma drugs [129]. LM22A-4 is a neurotrophic factor mimic encapsulated with Annexin V-conjugated cubosomes (L4-Acs) for administration to injured RGCs. The author demonstrated that L4-Acs with LM22A-4 conveyance may well be a useful approach to anticipate RGC misfortune in glaucoma patients. 

Despite icariin’s (ICA) activity against the growth of cancer, its clinical applicability is limited due to the destitute dissolvability in an aqueous environment. Fahmy and co-workers optimized and investigated the efficacy and mechanism of action of ICA-loaded cubosomes against ovarian cancer. ICA-loaded cubosomes (ICA-Cubs) demonstrated higher levels of apoptosis and cytotoxicity compared with ICA alone against ovarian cancer cell lines (Caov 3 and SKOV-3). However, ICA-Cubs demonstrated a non-cytotoxic impact on typical EA.hy926 endothelial cells. In the SKOV-3 cell line, it boosted the overexpression of p53, caspase-3, and the production of reactive oxygen species (ROS). However, ICA-Cubs also improved the efficacy of free drugs as it improved the solubility and cellular permeability of ICA [38]. 

Thymoquinone (TQ) is a bioactive compound of *Nigella sativa* that exhibits anticancer activity. The clinical application of TQ is hindered due to the lack of measurement strategies in the blood and tissues as well as its low bioavailability. Cubosomes are used to encapsulate the drug and deliver the anticancer molecule. A TQ-loaded compound formulation demonstrated enhanced antitumor activity as well as a dose-dependent reduction in treatment response. The usefulness of this nanosystem for TQ encapsulation was attributed to its high entrapment efficiency and zeta potential. TQ encapsulated in cubosomal nanoparticles is a promising technology for antitumour drug delivery that can be labelled, detected, and tracked within human cells [39].

### 5.5. Theranostic Application

Lipid nanoparticles have various functions. Recently, when the drugs have been loaded into LCNPs such as cubosomes, which act as drug carriers, such nanoparticles have been discovered to have combinatorial properties including the ability to enhance the therapeutic efficiency of the drugs against certain types of cancer cells as well as the capacity to diagnose a particular disease, also known as theranostic properties. This includes the in vivo imaging of both cells and molecules, molecular medicine therapeutics, image-guided therapeutics and microscopy, biosensors, and even customized or personalized treatment. In fact, LCNPs, particularly cubosomes, can permit the efficient loading of drugs due to their cavern-like structures. Thus, cubosomes are widely used as effective drug carriers via either the topical or subcutaneous route [130]. However, theranostic nanobioengineering has been implemented in the treatment of solid tumors. For instance, due to the small sizes of cubosomes, the drugs will be able to effectively permeate into the body as well as assemble precisely at the specific tumor sites without affecting other normal tissues and cells; this will eventually cause improved retention at those tumor sites. This is performed by using vascular fenestrations to passively target the particles to the site at which the tumor is located [130]. In addition, when substances such as polyelectrolytes are coated over the cubosomes, the initial burst release of the drugs can be prevented [40]. Other species can also be attached onto the surface of the nanoparticles in order to enhance their capability to target certain cells and to enable extensive modes of detection. Moreover, the diagnostic properties of nanoparticles can be achieved simultaneously with their therapeutic properties as medical imaging agents, including MRI and X-ray contrast agents and fluorescent compounds, can be loaded into cubosomes to aid in diagnosis and/or monitor the progress of therapy [131]. This is very important as it contributes to the development of multifunctional vehicles required for drug delivery. 

A recent study examined the therapeutic properties of cubosomes coated with polylisine, and the cubosomes were combined with two different hydrophobic anticancer medications (cisplatin and PTX). This study was conducted to investigate their therapeutic efficacy against cervical cancer cell lines, known as HeLa cells, and their cytotoxicity against human hepatoma cell lines, known as HepG2 [40]. In fact, both cisplatin and PTX are claimed to be effective and potent anticancer drugs, yet cancer cells can develop resistance to drugs by minimizing the absorption of drugs into cells and releasing the drugs to the outside of the cells. This could be because the structure of membrane transporters has changed, making it harder for drugs to pass through. However, there are possibilities of mutations that may occur, which would minimize the number of membrane transporters, eventually causing decreased drug absorption. Therefore, the use of cubosomes can be a better option, and the coated cubosomes will prevent the initial burst release of the drugs as well as allow sustained release of the drugs. The findings showed that the drugs were dispersed uniformly in the cubosomes after undergoing morphological analysis, differential scanning calorimetry, and X-ray diffraction studies. In terms of cytotoxicity, coated cubosomes were discovered to have a lower toxicity against HepG2 when compared with the uncoated ones, indicating that coating may decrease the cytotoxic effect of cubosomes. This could be due to faster drug release when uncoated cubosomes are added into the cell cultures, leading to more toxic effects on the cells. Furthermore, in terms of therapeutic efficiency, the coated cubosomes have higher therapeutic efficacy against HeLa cells when compared with the uncoated ones, as more destruction of the cells could be seen following fluorescence microscopy when coated cubosomes were infused. As a result, this study demonstrated the efficacy of drug-loaded cubosomes coated with polylisine in targeting cancer cells. 

Another in vitro study was carried out to identify the theranostic properties of hexosomes, also known as the dispersions of the reverse hexagonal phase. In addition, the action of stabilizing polymers contributes to the colloidal stability of hexosomes as their major roles are to promote steric stability against aggregation, although it may also help to reduce the rate at which plasma proteins eliminate nanostructures from the systemic circulation. For instance, Pluronics are the most widely utilized polymers for this purpose. Other stabilizing compounds such as DSPE-PEG2000 and Citrem can also be used. In this study, the anticancer drug docetaxel was incorporated into MO-based hexosomes and the nanoparticles were covered within a hydrophilic polymeric layer of Pluronic F108, which was both rhodamine- and folate-conjugated [41]. In fact, docetaxel is a chemotherapeutic drug with low water solubility as well as poor selectivity, which will cause unwanted side effects because of the destruction of cancer cells and healthy cells. Therefore, this system was prepared to increase its water solubility and bioavailability. However, folic acid as a targeting moiety and rhodamine as an imaging moiety were incorporated into the hexosomes, contributing to its theranostic properties in cancer treatment. The effect of drug-loaded hexosomes was investigated on HeLa cells. The results revealed that the docetaxel-loaded hexosomes were highly stable, as investigated by the in vitro release study, as the drug was released in a slow and continuous manner. Moreover, drug-loaded hexosomes showed a relatively higher cytotoxicity against HeLa cells when compared to the free drug alone as there was a significant decrease in the viability of HeLa cells caused by drug-loaded hexosomes. Furthermore, with the addition of fluorescent rhodamine, the extent of uptake of nanoparticles by the cells can be monitored. Thus, hexosomes with the incorporation of certain moieties can result in significant theranostic properties in the case of cancer treatment. 

### 5.6. Vaccine Delivery

Vaccines are claimed to be effective when they are able to promote strong humoral and cellular immune responses [132]. However, the immunogenicity of vaccines, such as protein- and peptide-based vaccines is one of the important challenges when it comes to vaccine delivery. It was previously questionable as to whether such subunit vaccines had the ability and effectiveness to potentiate immune responses, as previous studies had demonstrated a low potentiation of immune responses when vaccines were directly administered [63]. This may have been due to the lack of the secondary signals needed to stimulate immune responses of the pure proteins and peptides, eventually contributing to the low immunogenicity [133]. However, recent developments have ascertained that lipid-based particulate delivery systems such as LCNPs can be incorporated into vaccine delivery systems to enhance the body’s immune system. However, adjuvants such as Freund’s adjuvant and an aluminum adjuvant may be incorporated in clinical vaccines. Nevertheless, certain adjuvants can have disadvantages, including the emergence of adverse events such as local inflammation and a failure to promote a cell-mediated immunological response [134]. Recently, nanoparticles including liposomes, cubosomes, and PLGA have been used in the development of adjuvants, resulting in the greater potentiation of humoral as well as cellular immune responses when compared to the antigen alone. Furthermore, other adjuvants including the Toll-like receptor antagonist imiquimod, as well as monophosphoryl lipid A, have been added to the nanoparticulate system in recent attempts to develop effective compound-based vaccine delivery methods [132]. In addition, the potential of LCNPs in vaccine administration with improved immunogenicity has been investigated using ovalbumin (OVA) as a model subunit antigen. In fact, it is essential for ensuring the antigens and immune potentiators such as adjuvants present in a vaccine have the ability to interact with the body’s immune system and successfully boost the immunological response; hence, both antigens and adjuvants are claimed to be largely responsible for the vaccines’ effectiveness [43,44]. 

In a further study, a substance called ginseng stem-leaf saponin (GSLS) was demonstrated to have the potential to improve the immune response against diseases, particularly bivalent Newcastle disease [134]. In addition, the combination of GSLS and thimerosal can enhance the immunization of attenuated pseudorabies virus vaccines. Other than immunological effects, GSLS is also associated with properties such as anti-apoptotic, antitumor, and even hypoglycemic effects. Due to GSLS’s hydrophilicity and small molecular weight which result in its low bioavailability, it can be encapsulated in cubosomes as the cubosomes have been shown to have successful encapsulation efficiency and their presence can lead to the sustained release of drugs. Furthermore, due to the negative charges of cubosomes, which lead to bioactivity limitations, cationic modification such as the incorporation of chitosan can be carried out to solve the problem. Furthermore, due to the presence of chitosan, its gel matrix can be tightly bound to the antigen-containing cubosomes because of the opposite charges, leading to a slowdown in antigen release. For example, Qiu et al. have shown that the use of chitosan-modified GSLS-encapsulated cubosomes (Cub-GSLSCS), as carriers in delivering vaccines is due to their immunopotentiation as well as adjuvanticity [134]. The potential of Cub-GSLSCS to trigger an immune response was determined by potentiating macrophages in vitro, whereas its adjuvanticity was identified through the immune response triggered by the OVA model antigen. In this study, the encapsulation efficiency of the cubosomes was relatively high, indicating that a large number of antigens were successfully adsorbed onto the cubosomes after chitosan modification. It was also shown that the cubosomes had component colloidal stability, acceptable sustained-release property, as well as excellent physical stability. Cub-GSLSCS has been shown to increase the secretion of various cytokines such as interleukin-6, interleukin-12, and TNF- α, which may help macrophages become more activated and respond to cytokine release. However, this nanoparticle system can be used as an attractive adjuvant in vaccine delivery systems, as the results showed a significant increase in the CD4+/CD8+ T cell ratio when Cub-GSLSCS-OVA was administered, indicating the cellular immune response can be enhanced by producing OVA-specific IgG antibodies. 

Moreover, another study demonstrated the use of *Ganoderma lucidum* polysaccharide (GLP), which is one of the essential components present in this particular fungal species, in immunomodulation and its potential to be used as an adjuvant in vaccine delivery. Nonetheless, GLP lacks the capacity to target the antigen-presenting cells (APCs), limiting its use; hence, the potential of cubosomes was evaluated as they can encapsulate the antigen or drug in order to improve targeting and GLP’s adjuvanticity. In fact, in the case of subunit vaccines, surface engineering of the nanoparticles plays a significant role in altering their physicochemical characteristics, increasing vaccine-stimulated immune responses, and eventually resulting in a long-lasting immune reaction. For example, coating cubosomes with a single layer of polylisine improves their function as well as their anticancer properties. For instance, substances including cetyltrimethylammonium bromide (CTAB) and polydiallydimethyl ammonium chloride (PDDAC) were attached to the surface of GLP cubosomes (GLPC) in the aforementioned study, and their effects in targeting and activating the APCs were also investigated. Furthermore, the study showed the ability of OVA antigens adsorbed on CTAB-GLPC and PDDAC-GLPC nanoparticles to produce humoral and cellular immune responses. According to the findings, PDDAC modification aided in the maturation of dendritic cells and the uptake of antigens. However, PDDAC-GLPC-OVA was able to maximize the proliferation of T-cells such as CD3+ CD8+ or CD3+ CD4+, which further increased B-cell activation. As measured by enzyme-linked immunosorbent assay on the 14th day after the final vaccination, PDDAC-GLPC-OVA also successfully produced more cytokines such as interleukin-4, interleukin-6, IFN-γ, and TNF-α. In fact, the maturation of dendritic cells into draining lymph nodes, stimulation of the spleen, and the production of various cytokines into the systemic circulation may all contribute to the enhancement of immune responses caused by PDDAC-modified GLPC-OVA. Based on the findings, PDDAC alteration can improve humoral and cellular immune responses, indicating that it may be a potential protein-antigen vaccine adjuvant [132]. 

### 5.7. Challenges and Outlook

The use of LCNPs as a drug delivery system has several challenges. One such limitation is the presence of a significant quantity of water within the system, which poses a challenge for the loading of hydrophilic drugs into the bilayer mesophase. Furthermore, the small dimensions of the particles provide a limited extent of diffusion pathways, which pose a challenge in regulating the hydrophilic drug release kinetics. Moreover, techniques for characterizing LCNPs are costly and challenging, necessitating complex protocols. The inherent variability of crystalline structures poses a significant challenge to their characterization. A minor alteration in a parameter, such as temperature or pressure, during a process can led to the formation of a product exhibiting a distinct crystal structure. Hence, it is imperative to employ suitable optimization techniques for the preparation process. The optimization of these processes can incur significant expenses, particularly when considering the scaling up necessary for industrial production. Furthermore, the inverse bicontinuous cubic phases and hexagonal phases exhibit significant viscosity, which impairs their suitability as parenteral dosage forms due to their mechanical rigidity. Increased fluid volumes can result in phase transitions, thereby causing undesirable rates of drug release. Furthermore, it is important to understand that the inclusion of therapeutic compounds, alteration of the amphiphilic lipid, and surfactants in LCNPs would inevitably impact their toxicity. Therefore, it is necessary to conduct formulation optimization and toxicity assessments to ensure the biocompatibility of LCNPs. Despite several challenges, LCNPs are still being highly explored as drug delivery carriers owing to their versatility and ability to maintain drug stability for prolonged periods, as compared with other nanoparticles. The ability to accommodate drugs with diverse properties, ease of handling, and reduced toxicity makes LCNPs superior in comparison to other lipid-based dosage forms. 

## 6. Conclusions

In conclusion, self-assembled LCNPs, particularly cubosomes and hexosomes, can be widely used in various fields, especially in the pharmaceutical sector. In this review, we have included the structures of both cubosomes and hexosomes and their characterization techniques. We also discussed the materials and methods involved in the preparation of LCNPs. Recent development has been established regarding the use of LCNPs as drug carriers in numerous drug delivery systems such as oral, transdermal, brain, vaccine, as well as targeted delivery. However, LCNPs are also well-known for their theranostic properties. The benefits of incorporating LCNPs as drug carriers into drug delivery systems include enhancing drug solubility, improving drug delivery, sustaining and controlling the release of drugs, enhancing drug permeation, improving the therapeutic effectiveness of drugs, and even enhancing the immune cellular response. In fact, the fate of these advantages is dependent on the physicochemical properties of LCNPs, where it might be affected by the size of nanoparticles or the surface potential of nanoparticles. Moreover, the biological factors such as first-pass metabolism or the BBB, will also affect drug delivery to the desired part of the body. 

Nevertheless, characterization techniques for LCNPs are often costly and complicated owing to the dynamic crystal properties, which also bring about challenges in producing LCNPs where the crystal structure can be altered upon slight changes in the pressure or temperature, forming another product. Hence, the manufacturing process needs to be optimized. Moreover, long-term stability is necessary in the effort of translating LCNPs into clinical use; therefore, further formulation developments are required to produce stable formulations of LCNPs with an extended shelf-life. However, the applications of LCNPs in clinical use are still questionable due to the limitations of the clinical trial evidence. Challenges such as the long-term stability of nanoparticles, bioavailability, efficacy of targeting, and toxicity should be addressed before these drug delivery systems are incorporated in clinical trials. Therefore, these findings warrant further exploration and investigation in terms of the development of drug delivery systems using LCNPs. 

## Figures and Tables

**Table 1:** Summary of various applications of LCNP systems and their respective advantages.

Types of LCNP	Applications	Therapeutic Molecules	Advantages	References
**Cubosomes**	Oral delivery	Cyclosporin A	Improved solubility, protection from harsh environments and enzymatic degradation	[11,12]
		Simvastatin		
		Curcumin	Improved cytotoxic activities	[13]
		Doxorubicin	Improved antitumor efficacy and bioavailability, reduced risk of cardiotoxicity	[14]
**Cubosomes**		CoQ_10_	Improved drug delivery, enhanced hepatoprotective effect	[15]
		Amphotericin B	Improved drug delivery	[16]
		Gliclazide	Improved bioavailability, increased therapeutic effect	[17]
	Topical (eyes)	Ketoconazole	Improved antifungal action, enhanced drug permeability	[18]
		Latanoprost	Enhanced effectiveness, improved sustained-release property	[19]
		Beclomethasone dipropionate	Enhanced effectiveness	[20]
	Topical (ears)	Norfloxacin	Enhanced permeation and accumulation of drugs at specific site	[21]
	Transdermal	Capsaicin	Improved sustained skin retention and sustained-release property	[22]
		Rapamycin	Enhanced efficiency and sustained-release property	[23]
		Tenoxicam	Enhanced effectiveness	[24]
		Colchicine	Increased drug absorption	[25]
		Dapsone	Enhanced skin permeation	[26]
**Cubosomes and Hexosomes**	Brain (IV)	Phenytoin	Enhanced brain penetration of blood–brain barrier	[27]
**Cubosomes**		RhoB	Enhanced uptake of drug	[28]
		Doxorubicin and loperamide	Increased drug delivery	[29]
		S14G-HN peptide	Increased drug delivery and effectiveness	[30]
	Targeted delivery route	Antimicrobial peptides	Enhanced penetration of LPS layers	[31]
		Dapsone	Increased permeation of drug	[32]
		Paclitaxel	Enhanced controlled release and cellular uptake	[33]
		Fluoxetine hydrochloride	Prolonged in vitro drug release	[34]
		Cisplatin	Better cytotoxic impact	[35]
		Albendazole	Increased bioavailability of drug	[36]
		Sorafenib	Increased cellular absorption and therapeutic anticancer activity	[37]
		Icariin	Improved solubility and cellular permeability	[38]
		Thymoquinone	Encapsulated drug and delivers anticancer molecule	[39]
**Cubosomes**	Theranostic	Cisplatin (coating: polylisine)	Prevents initial burst release of drugs/higher therapeutic efficacy	[40]
		Paclitaxel (coating: polylisine)	Prevents initial burst release of drugs/higher therapeutic efficacy	[40]
**Hexosomes**	Theranostic	Docetaxel	Higher cytotoxicity against certain cell lines and able to monitor the extent of nanoparticle uptake	[41]
**Cubosomes**	Vaccine	Polysaccharide, promising adjuvant for vaccines	Enhanced ability of immunostimulants to generate an immune response	[42]
		Ovalbumin	Producing nanoparticulate vaccine formulations in dry powder form	[43]
		Ovalbumin (OVA) absorbed cetyltrimethylammonium bromide-modified polygonatum sibiricum polysaccharide cubosomes	Stimulates the cellular immune response and increases the level of humoral immunity	[44]

## Data Availability

Not applicable.

## Abbreviations

a, average lattice parameter; $a_{0}$, effective headgroup area; *ABZ*, albendazole; AD, Alzheimer’s disease; AMP, *antimicrobial peptide*; AMPK, AMP-activated protein kinase; AMPs, antibiotic peptides; APCs, antigen-presenting cells; Au, gold; BBB, blood–brain barrier; Caov-3, ovarian cancer cell line; CNS, central nervous system; CoQ_10_, coenzyme Q_10_; CPP, critical packing parameter; cryo-FESEM, cryo-field emission scanning electron microscopy; cryo-TEM, cryo-electron microscopy; CSF, cerebrospinal fluid; CT, computed tomography; CTAB, cetyltrimethylammonium bromide; Cub-GSLSCS, GSLS-encapsulated cubosomes; d, interplanar spacing; D, Schwarz diamond (*Pn*3*m*); Da, Dalton; DLS, dynamic light scattering; DNA, deoxyribonucleic acid; DSPE-PEG_2000_, 1,2-distearoyl-sn-glycero-3-phosphoethanolamine-N-[methoxy(polyethylene glycerol)-2000]; EGFRs, epithelial growth factor receptors; EPR, enhanced permeation and retention; *Fd*3*m*, inverse discontinuous micellar cubic; FH, fluoxetine hydrochloride; G, Schoen gyroid (*Ia*3*d*); GDO, glycerol dioleate; GIT, gastrointestinal tract; GLP, *Ganoderma lucidum* polysaccharide; GLPC, GLP cubosomes; GMO, glycerol monooleate; GSLS, ginseng stem-leaf saponin; H_II_, hexagonal phase; HCT-116, human colorectal carcinoma cell line; HepG2, hepatoma cell line; I_2_, inverse discontinuous micellar cubic phase; ICA, icariin; ICA-Cubs, ICA-loaded cubosomes; IFN-γ, interferon-gamma; IOP, intraocular pressure; $l_{c}$, effective hydrophobic chain length; L_α_, lamellar phase; L_2_, inverse micelles; L_3_, swollen sponge phase; LC, liquid crystalline; LCNPs, liquid crystalline nanoparticles; LDH, *lactate dehydrogenase;* LL-37, human cationic antibacterial protein; LPS, lipopolysaccharide; MIC, minimal inhibitory concentration; MO, monoolein; MRI, magnetic resonance imaging; mTOR, mammalian target of rapamycin; NADPH, nicotinamide adenine dinucleotide phosphate; NPs, nanoparticles; N-to-B, nose-to-brain; OVA, ovalbumin; P, primitive (*Im*3*m*); PBPK, physiologically based pharmacokinetic modelling; PC, phosphatidylcholine; PDDAC, poly-diallydimethyl ammonium chloride; PEO, polyethylene glycol; P-gp, P-glycoprotein; PHYT, phytantriol; PLGA, poly (lactide-co-glycolide)/poly (lactic-co-glycolic acid); PLM, polarized light microscopy; PPO, polypropylene glycol; PTX, paclitaxel; q, scattering vector; QSAR, quantitative structure–activity relationship; RGCs, retinal ganglion cells; RhoB, lissamine rhodamine; ROS, reactive oxygen species; S14G-HN, Ser14Gly-humanin; SANS, small-angle neutron scattering; SAXS, small-angle X-ray scattering; SLN, solid lipid nanoparticles; SMO, sorbitol monooleate; SPECT, single-photon emission computed tomography; TA, tocopherol acetate; TAA, thioacetamide; TNF-α, tumor necrosis factor alpha; TQ, thymoquinone; v, volume of the hydrophobic chain; V_II_, inverse bicontinuous cubic phase; WAXS, wide-angle X-ray scattering; XRD, X-ray diffraction; 99mTc, technetium-99 m.
